# Exploring research hotspots and emerging trends in neuroimaging of vascular cognitive impairment: a bibliometric and visualized analysis

**DOI:** 10.3389/fnagi.2024.1408336

**Published:** 2024-07-08

**Authors:** Fangyuan Xu, Ziliang Dai, Wendong Zhang, Yu Ye, Fan Dai, Peijia Hu, Hongliang Cheng

**Affiliations:** ^1^The First Clinical Medical School, Anhui University of Chinese Medicine, Hefei, China; ^2^Department of Rehabilitation Medicine, The Second Hospital of Wuhan Iron and Steel (Group) Corp., Wuhan, China; ^3^Department of Neurology, The Second Affiliated Hospital of Anhui University of Chinese Medicine, Hefei, China; ^4^The Second Clinical Medical School, Anhui University of Chinese Medicine, Hefei, China; ^5^Department of Endocrinology, The Second Affiliated Hospital of Anhui University of Chinese Medicine, Hefei, China

**Keywords:** vascular cognitive impairment, neuroimaging, bibliometric analysis, research hotspots, research trends

## Abstract

**Background:**

Vascular cognitive impairment (VCI) manifests in memory impairment, mental slowness, executive dysfunction, behavioral changes, and visuospatial abnormalities, significantly compromising the quality of daily life for patients and causing inconvenience to caregivers. Neuroimaging serves as a crucial approach to evaluating the extent, location, and type of vascular lesions in patients suspected of VCI. Nevertheless, there is still a lack of comprehensive bibliometric analysis to discern the research status and emerging trends concerning VCI neuroimaging.

**Objective:**

This study endeavors to explore the collaboration relationships of authors, countries, and institutions, as well as the research hotspots and frontiers of VCI neuroimaging by conducting a bibliometric analysis.

**Methods:**

We performed a comprehensive retrieval within the Core Collection of Web of Science, spanning from 2000 to 2023. After screening the included literature, CiteSpace and VOSviewer were utilized for a visualized analysis aimed at identifying the most prolific author, institution, and journal, as well as extracting valuable information from the analysis of references.

**Results:**

A total of 1,024 publications were included in this study, comprising 919 articles and 105 reviews. Through the analysis of keywords and references, the research hotspots involve the relationship between neuroimaging of cerebral small vessel disease (CSVD) and VCI, the diagnosis of VCI, and neuroimaging methods pertinent to VCI. Moreover, potential future research directions encompass CSVD, functional and structural connectivity, neuroimaging biomarkers, and lacunar stroke.

**Conclusion:**

The research in VCI neuroimaging is constantly developing, and we hope to provide insights and references for future studies by delving into the research hotspots and frontiers within this field.

## 1 Introduction

Vascular cognitive impairment (VCI) refers to cognitive deficits arising from various vascular causes like clinical cerebral infarction, brain hemorrhages, and subclinical vascular brain damage (Gorelick et al., 2011; Rundek et al., 2022). VCI encompasses the spectrum of different degrees of cognitive dysfunction, ranging from mild cognitive impairment to vascular dementia (VaD) or mixed vascular and other types of dementia (Farooq et al., 2017). It can manifest in memory impairment, mental slowness, executive dysfunction, behavioral changes, and visuospatial abnormalities (Kalaria, 2016; van der Flier et al., 2018), significantly compromising the quality of daily life for patients and causing inconvenience to caregivers. Notably, cardiovascular risk factors such as smoking, hypertension, high cholesterol, and diabetes mellitus at midlife contribute to a 20% to 40% heightened risk of developing dementia in late life (Whitmer et al., 2005). Therefore, early intervention targeting these factors may lower future dementia risks. The broad range of VCI was defined in various terms, thus posing challenges in tracking the epidemiological data (Farooq and Gorelick, 2013). Vascular dementia (VaD), comprising 15—30% of dementia cases, is the second most prevalent subtype after Alzheimer’s disease (AD) (Wolters and Ikram, 2019). A study demonstrated that annual costs for VaD patients exceed those for AD (Fillit and Hill, 2002), underscoring the need for early diagnosis, intervention, and treatment to improve the prognosis of patients and alleviate substantial economic burdens.

Neuroimaging serves as a crucial approach to evaluating the extent, location, and type of vascular lesions in patients suspected of VCI. Both computed tomography (CT) and magnetic resonance imaging (MRI), widely applied imaging modalities in clinical practice, detect vascular lesions and structural abnormalities (Zukotynski et al., 2018). Nevertheless, MRI is the preferred modality for assessing VCI due to its high sensitivity and specificity. Its diverse sequences mainly involve T1-weighted imaging to detect global atrophy, T2-weighted imaging for identifying lacunar infarcts, susceptibility-weighted imaging (SWI) for microbleeds detection, and the fluid-attenuated inversion recovery (FLAIR) sequence for detecting white matter lesions (van der Flier et al., 2018). Positron emission tomography (PET) encompasses F-fluorodeoxyglucose (FDG)-PET, Amyloid PET, and Tau PET, which assist in the identification of various dementia types (Frantellizzi et al., 2020). Single photon emission computed tomography (SPECT) is widely used in dementia patients. It quantifies cerebral perfusion, contributing to the differentiation between vascular diseases and AD (Frantellizzi et al., 2021). Despite the substantial volume of publications on neuroimaging in VCI, a comprehensive analysis to explore research hotspots and future trends in VCI neuroimaging is notably lacking.

Bibliometric analysis employs mathematical and statistical methodologies to quantitatively analyze academic literature within a specific field. By delving into the cooperation relationships among authors, countries, and institutions, along with the citation analysis of references, elucidating the current development status, identifying research hotspots, and future research directions in that domain. Furthermore, the results of bibliometric analysis can manifest not only in a narrative review but also in visual network maps. To our knowledge, a comprehensive and in-depth bibliometric analysis pertaining to VCI neuroimaging is currently lacking. Therefore, we utilized two widely used software programs, CiteSpace and VOSviewer, for conducting a bibliometric analysis. The primary objective of this article is to identify the hotspots and emerging frontiers of VCI neuroimaging and provide insightful information for further research on this topic.

## 2 Materials and methods

### 2.1 Literature source and data retrieval methods

**FIGURE 1:**
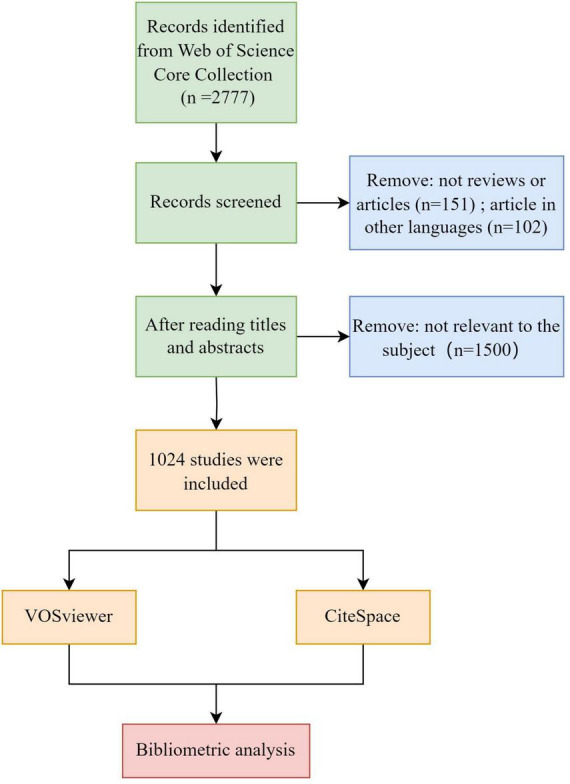
The flow diagram for the literature screening.

The included literature was retrieved from the Core Collection of the Web of Science, spanning from January 1, 2000, to August 31, 2023. The retrieval strategy involved identifying theme words related to neuroimaging and VCI. The specific retrieval formula is shown in Supplementary Table 1, and only articles and reviews pertinent to the research topic will be included for subsequent analysis. Furthermore, publications were restricted to those in English. After comprehensive searching and screening (refer to Figure 1), a total of 1,024 publications, comprising 919 articles and 105 reviews, were selected for this bibliometric analysis. Subsequently, we exported the details containing complete records and cited references in the form of plain text files.

### 2.2 Data analysis

VOSviewer V1.6.19 and CiteSpace V6.2.R3, both Java-based programs, were useful tools for performing bibliometric and visualized analyses. VOSviewer can extract crucial information from included articles to establish collaborative, co-occurrence, and co-citation networks. It calculates similarity matrices by analyzing co-occurrence matrices, subsequently employing the VOS mapping technique to generate a distance-based visualization map (van Eck and Waltman, 2010; Qu et al., 2024). In this study, VOSviewer was applied to illustrate and explore collaborative relationships among authors, countries, and organizations, as well as the co-occurrence networks of keywords and the visualization networks of journals and co-cited journals. Node size denotes the frequency of occurrence, while the thickness of links between nodes depicts connection strength, with nodes automatically clustered and represented in distinct colors. The total link strength is calculated to manifest the cumulative strength of links between two nodes in a visualization network map. In addition, time factors are considered in the overlay visualization analysis of keywords. Nodes of different colors represent different years of keyword occurrences, with each node’s color determined by the average year of the keyword’s appearance.

We employed CiteSpace (Chen, 2006) for clustering analyses of keywords and references, which facilitated a deeper understanding of the research hotspots in VCI neuroimaging. Our analysis was performed with the following parameters: time slicing (2000–2023), 1 year per slice, link strength (cosine), the selection of each slice (g-index with *k* = 25 as the scale factor), and pruning (pathfinder and pruning the merged network). Clustering analysis utilized the log-likelihood ratio (LLR) algorithm, with each cluster labeled and sorted to elucidate research status and hotspots. A Modularity Q value exceeding 0.3 indicates a significant clustering structure, while a weighted mean silhouette > 0.5 signifies the reliability of the clustering results. Moreover, the identification of keywords and references with strong citation bursts facilitates predicting emerging trends and research frontiers.

## 3 Results

### 3.1 Publications outputs and trends

**FIGURE 2:**
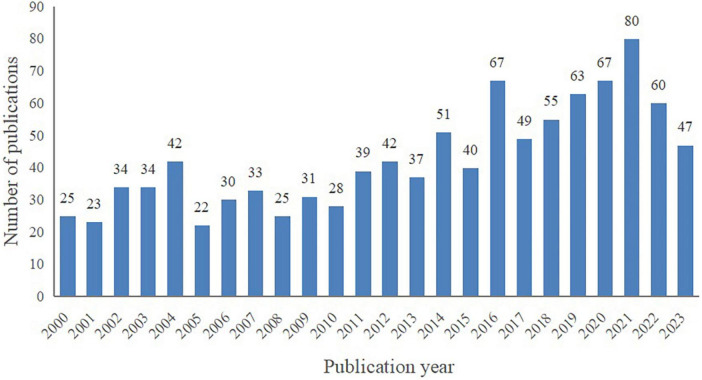
The annual publication trend map of the included literature from 2000 to 2023.

A total of 1,024 publications spanning from January 2000 to August 2023 were included in this bibliometric analysis, consisting of 919 articles (89.75%) and 105 reviews (10.25%). As depicted in Figure 2, despite certain fluctuations, the number of publications on VCI neuroimaging shows an overall upward trend. Notably, marked increases were observed in 2014 and 2016, and 2021 was the peak year with 80 publications. These findings indicate the growing attention researchers are devoting to VCI neuroimaging, and further studies are required to advance its development.

### 3.2 Analysis of core authors

**FIGURE 3:**
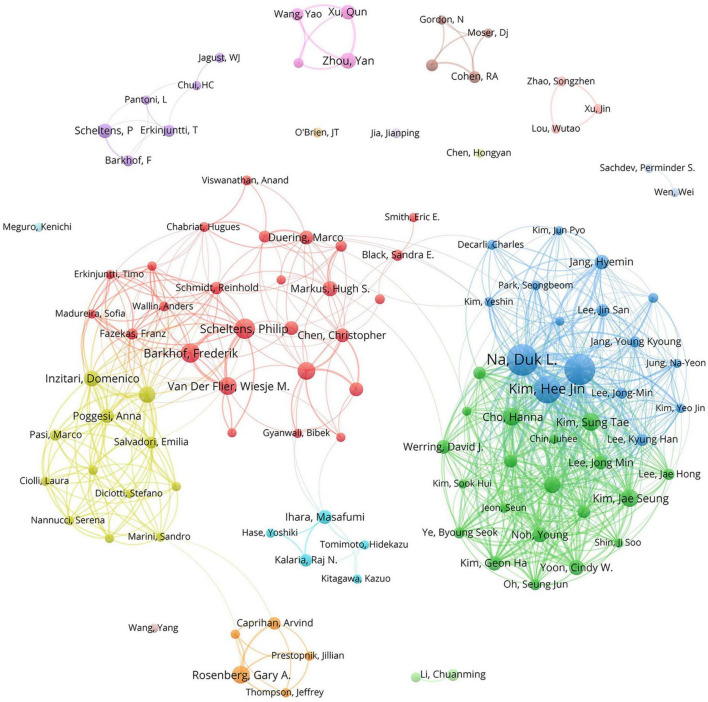
The collaboration network map of core authors with at least six publications. The size of the nodes indicates the number of publications, and the thickness of the links between the nodes represents the strength of collaborative relationships among core authors. Distinct colors are used to distinguish different clusters, and the cooperative relationships between nodes in the same cluster are relatively close.

The 1,024 retrieved literature involved 5,404 authors. According to the scholar Price’s law (Price, 1963), the minimum publication volume for core authors is *m* = 0.749 × $\sqrt{n_{\text{max}}}$, where n_max_ represents the number of published articles conducted by the most prolific author (n_max_ = 50 here). Hence, the core authors should have released at least six papers. By the calculation of VOSviewer, there are 110 core authors, accounting for 2.04% of the total number of authors. Na, Duk L. emerged as the most prolific author with 50 studies and 1,682 citations. His research concentrated on the role of white matter hyperintensity in cognitive decline, the impact of cerebral amyloid burden on blood viscosity and cognition in subcortical VCI, as well as hippocampal changes, cortical thinning, and microbleeds in subcortical VaD (Seo et al., 2007; Kim et al., 2012, 2015, 2020; Lee et al., 2014; Noh et al., 2014). As listed in Table 1, Scheltens, Philip received the highest average number of citations, manifesting the influence and recognition of his articles among scholars. To facilitate a more intuitive observation of the collaborative relationships of the core authors, VOSviewer was utilized for network visualization analysis. Figure 3 shows that relatively stable cooperative groups have been formed among the core authors.

**TABLE 1:** The top 10 core authors in terms of publications.

Rank	Author	Documents	Citations	Average citation
1	Na, Duk L	50	1682	33.64
2	Seo, Sang Won	49	1674	34.16
3	Kim, Hee Jin	39	1214	31.13
4	Scheltens, Philip	24	1489	62.04
5	Barkhof, Frederik	21	1019	48.52
6	Kim, Sung Tae	19	808	42.53
7	van der flier, Wiesje M	19	864	45.47
8	Kim, Jae Seung	18	648	36
9	Cho, Hanna	18	671	37.28
10	Rosenberg, Gary A	18	794	44.11

### 3.3 Analysis of the collaboration of countries and institutions

**FIGURE 4:**
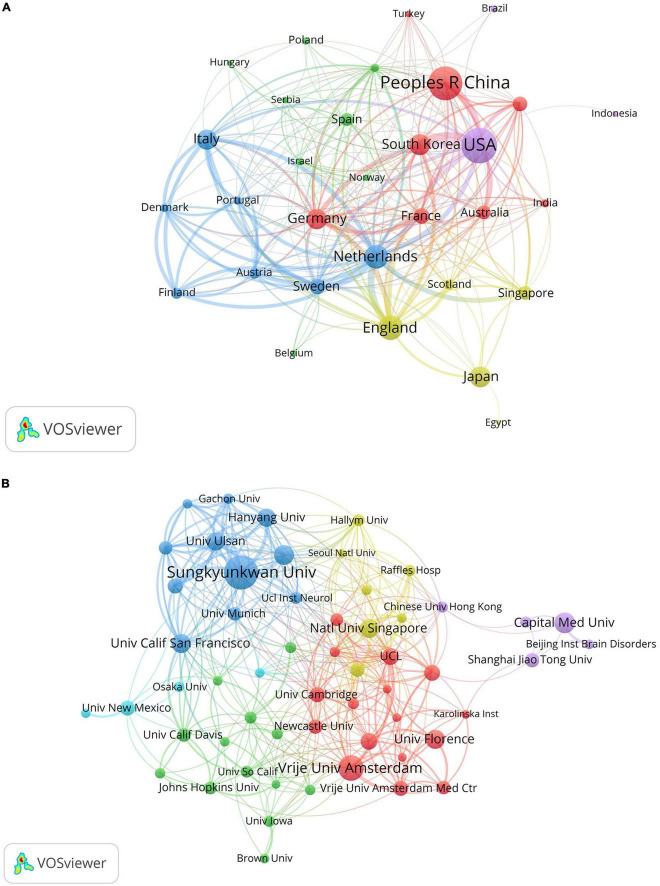
**(A)** The cooperation network map of countries about VCI neuroimaging; **(B)** the collaboration network map of institutions about VCI neuroimaging. The node sizes correspond to the volume of publications, while the link thickness indicates the collaborative strength. Different clusters are manifested by different colors, and the cooperative relationships between nodes in the same cluster are relatively close.

Analyzing information extracted from publications regarding countries and institutions can help understand their research contributions and collaborative relationships in VCI neuroimaging. Among the 1,024 retrieved articles, 60 countries and 1,431 institutions were involved. Table 2 indicates the USA as the most prolific country, contributing 239 studies, followed by China (212 studies) and England (119 studies). What’s more, the total link strength reflects the collaboration intensity between nations and organizations. The Netherlands exhibits the highest link strength (221), manifesting that the Netherlands has maintained closed connections with other countries in VCI neuroimaging research. Furthermore, Figure 4A displays a cooperation network map of countries with more than five publications. Regarding institutions, Sungkyunkwan University led in published articles (57 papers), followed by Vrije Universiteit Amsterdam (39 papers) and Capital Medical University (29 papers). Remarkably, Sungkyunkwan University also had the highest link strength (239). As shown in Figure 4B, different clusters and cooperation networks have been formed. Intriguingly, the majority of institutions on the top ten list were universities rather than hospital radiology departments.

**TABLE 2:** The top 10 countries and institutions in terms of frequency.

Rank	Country	Frequency	Total link strength	Institution	Frequency	Total link strength
1	USA	239	204	Sungkyunkwan University	57	239
2	Peoples R China	212	79	Vrije Universiteit Amsterdam	39	85
3	England	119	179	Capital Medical University	29	46
4	Netherlands	112	221	Samsung Medical Center	28	109
5	South Korea	86	95	University of California San Francisco	27	122
6	Japan	83	23	National University of Singapore	27	101
7	Italy	77	118	University of Florence	27	78
8	Germany	76	164	University of Ulsan	25	142
9	Sweden	48	117	Hanyang University	25	125
10	France	47	83	University College London	22	89

### 3.4 Analysis of journals and co-cited journals

**FIGURE 5:**
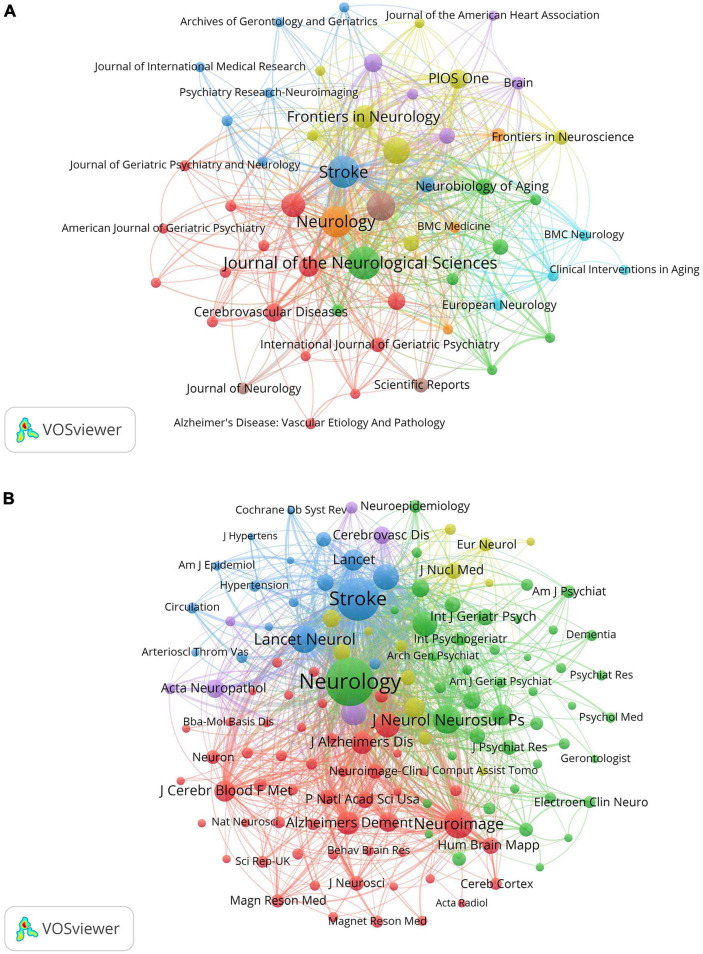
**(A)** The network visualization map of journals about VCI neuroimaging. The size of the node displays the number of publications in the journals, while the links between nodes denote the connection strength between the two journals. **(B)** The visualization map of co-cited journals about VCI neuroimaging. The node size signifies the co-citation frequency of the journals, and the links represent the co-citation relationship between journals. Journals within the same cluster exhibit relatively close co-citation relationships.

Table 3 lists the top ten journals in VCI neuroimaging, with the *Journal of the Neurological Sciences* leading with 50 articles, followed closely by *Stroke* (46 articles) and *Neurology* (45 articles). Notably, the 2022 impact factor (IF) of these top ten journals ranged from 2.4 to 11.1, with four being classified as Q1 in the JCR partition. A network visualization map of journals with more than five papers was conducted by VOSviewer and displayed in Figure 5A. In terms of co-cited journals, *Neurology*, *Stroke*, and *Neuroimage* emerged as the most frequently cited journals with 4,637, 3,369, and 1,281 citations, respectively. This visualization map is depicted in Figure 5B, where each node indicates a co-cited journal, categorized into different clusters distinguished by colors. Additionally, most of the top ten co-cited journals belong to the Q1 partition.

**TABLE 3:** The top 10 journals and co-cited journals with frequency and citation counts.

Rank	Journal	Frequency	2022 JCR partition (IF)	Co-cited journal	Citations	2022 JCR partition (IF)
1	Journal of the Neurological Sciences	50	Q2 (4.4)	Neurology	4637	Q1 (10.1)
2	Stroke	46	Q1 (8.4)	Stroke	3369	Q1 (8.4)
3	Neurology	45	Q1 (10.1)	Neuroimage	1281	Q1 (5.7)
4	Journal of Alzheimers Disease	38	Q2 (4)	Journal of Neurology, Neurosurgery and Psychiatry	1180	Q1 (11.1)
5	Frontiers in Aging Neuroscience	32	Q2 (4.8)	Lancet Neurology	1113	Q1 (48)
6	Dementia and Geriatric Cognitive Disorders	26	Q3 (2.4)	Brain	953	Q1 (14.5)
7	Frontiers in Neurology	25	Q2 (3.4)	Annals of Neurology	907	Q1 (11.2)
8	Journal of Neurology, Neurosurgery and Psychiatry	19	Q1 (11.1)	Journal of the Neurological Sciences	895	Q2 (4.4)
9	Plos One	17	Q2 (3.7)	Neurobiology of Aging	856	Q2 (4.2)
10	European Journal of Neurology	16	Q1 (5.1)	Alzheimers & Dementia	689	Q1 (14)

### 3.5 Analysis for clustering and citation bursts of keywords

**FIGURE 6:**
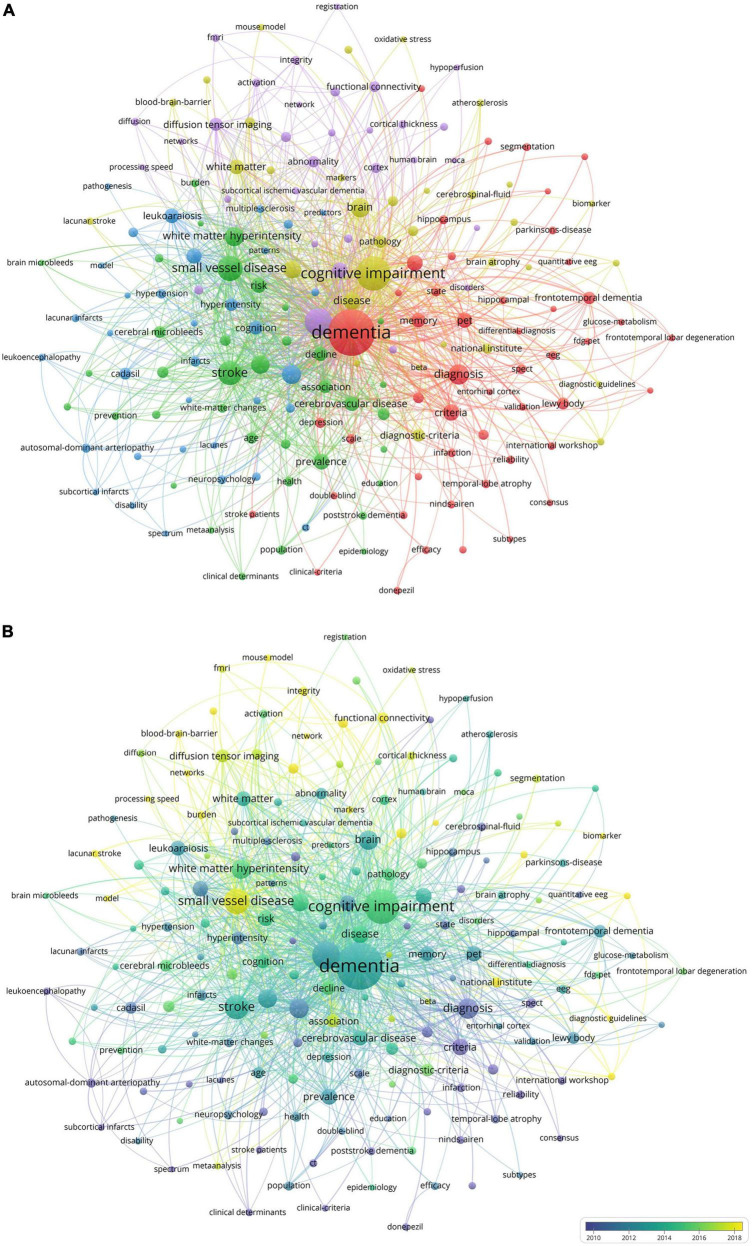
**(A)** The co-occurrence and clustering map of keywords about VCI neuroimaging. The node size indicates the frequency of keyword occurrences, and the links depict the co-occurrence relationship between two keywords. Keywords that are similar or closely related are classified into the same cluster. **(B)** The overlay visualization map of keywords about VCI neuroimaging. The different colors of nodes represent the appearance years of keywords, with purple and blue appearing earlier and green and yellow appearing later.

**FIGURE 7:**
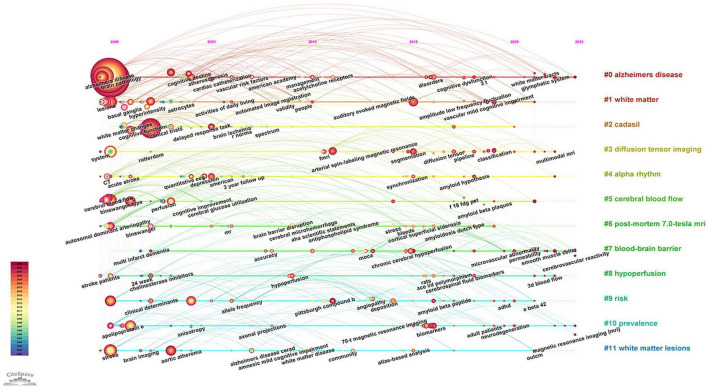
The timeline map of keywords about VCI neuroimaging. The right side of the map depicts twelve keyword clusters, each distinguished by a unique color, while the left side illustrates the temporal distribution of keywords within each cluster. Node size reflects the frequency of keyword occurrences.

Keywords are concise summaries of an article and facilitate readers grasping the core content in a short time. As detailed in Table 4, the most frequently appearing keywords are “dementia” (840 times), also presenting the highest total link strength (5,873), followed by “cognitive impairment” (424 times), “magnetic resonance imaging” (264 times), “small vessel disease” (216 times), and “stroke” (191 times). Figure 6A shows the clustering of keywords, with the largest red cluster mainly containing dementia, diagnostic criteria, and PET, while the green cluster primarily encompassing small vessel diseases, stroke, and white matter hyperintensity. Additionally, the purple cluster comprises functional connectivity, cortex, and diffusion tensor imaging, whereas the yellow clustering mainly includes cognitive impairment, brain, and pathology. In Figure 6B, the purple and blue nodes appear earlier and mainly encompass keywords like diagnostic criteria, dementia, magnetic resonance imaging, and stroke. Conversely, the green and yellow nodes emerge relatively later, involving small vessel diseases, functional connectivity, integrity, and diffusion tensor imaging, which also provide references for predicting future research directions. The timeline map of keywords (Figure 7) depicts the development context and evolution trend of research topics. Prior to 2010, the study mainly focused on cerebral blood flow, stroke, white matter changes, and Binswanger’s disease. There was an extensive exploration of neuroimaging techniques, including artistic spin labeling magnetic resonance, 7T MRI, diffusion tensor imaging, and ^18^F-FDG-PET following 2010.

**TABLE 4:** The top 10 keywords with frequency.

Rank	Keyword	Frequency	Total link strength
1	Dementia	840	5873
2	Cognitive impairment	424	3148
3	Magnetic resonance imaging	264	2093
4	Small vessel disease	216	1744
5	Stroke	191	1513
6	Diagnosis	134	971
7	Stroke	170	1457
8	White matter lesions	115	941
9	White matter hyperintensity	114	977
10	Brain	114	827

**FIGURE 8:**
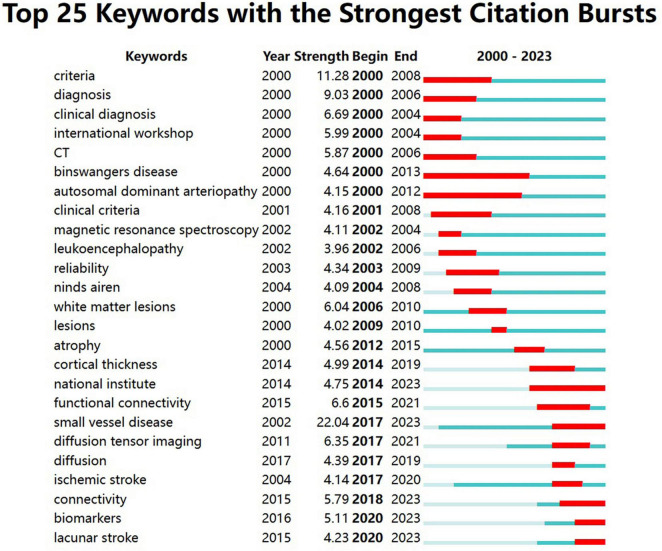
The top 25 keywords with the strongest citation bursts from 2000 to 2023. The burst detection can identify a surge in the citation frequency of keywords. The dark blue bar represents the time period of keyword appearance, while the red bar indicates the time period from the beginning to the end of the keyword burst.

Keyword bursts indicate a rapid increase in the frequency of keyword occurrences over a period of time. It offers insights into research hotspots and emerging frontiers within a specific field. The significance of keywords is demonstrated through their burst strength. As presented in Figure 8, the top three keywords with the strongest burst strengths are “small vessel disease” (2017–2023), “criteria” (2000–2008), and “diagnosis” (2000–2006). Keywords with the latest citation burst, including small vessel disease, connectivity, biomarkers, and lacunar stroke, hold potential for predicting future research directions in VCI neuroimaging.

### 3.6 Analysis of co-cited reference

**FIGURE 9:**
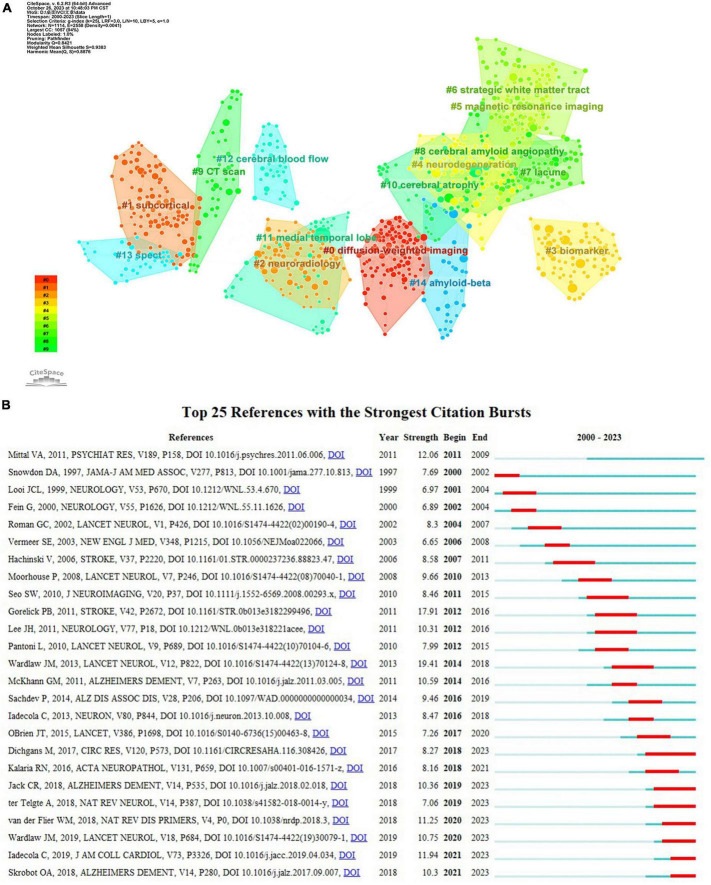
**(A)** The clustering map of co-cited references about VCI neuroimaging. The largest 15 clusters were displayed in different colors. **(B)** The top 25 references with the strongest citation bursts from 2000 to 2023. The burst detection can identify a substantial increase in the citation frequency of the references. The red bar highlights the time period of a reference citation burst.

Co-citation analysis facilitates the identification of a knowledge base and influential publications, allowing for further exploration of the research field. As listed in Table 5, the “Diagnostic and statistical manual of mental disorders” issued by the American Psychiatric Association in 1994 had the highest co-citation count with 72 times and the largest centrality (0.42). It was primarily concerning the diagnostic criteria of VaD. The second most frequently co-cited reference, exhibiting the strongest burst strength (see Figure 9B), was about the neuroimaging standard of small vessel disease (SVD). This article comprehensively analyzes and standardizes the definitions and terminologies of recent small subcortical infarcts, lacune and white matter hyperintensity of presumed vascular origin, perivascular space, cerebral microbleed, and brain atrophy. Moreover, it provides suggestions for the selection of neuroimaging approaches, imaging analysis, and reporting standards for SVD (Wardlaw et al., 2013b).

**TABLE 5:** The top 10 co-cited references with co-citation counts.

Rank	Co-citation counts	Title	References
1	72	Diagnostic and statistical manual of mental disorders	American Psychiatric Association, 1994
2	45	Neuroimaging standards for research into small vessel disease and its contribution to ageing and neurodegeneration	Wardlaw et al., 2013b
3	38	Vascular contributions to cognitive impairment and dementia: a statement for healthcare professionals from the American heart association/American stroke association	Gorelick et al., 2011
4	24	NIA-AA Research Framework: Toward a biological definition of Alzheimer’s disease	Jack et al., 2018
5	23	Vascular cognitive impairment	van der Flier et al., 2018
6	22	Identification of pure subcortical vascular dementia using 11C-Pittsburgh compound B	Lee et al., 2011
7	22	Small vessel disease: mechanisms and clinical implications	Wardlaw et al., 2019
8	22	Vascular Cognitive Impairment and Dementia: JACC Scientific Expert Panel	Iadecola et al., 2019
9	20	Vascular Cognitive Impairment	Dichgans and Leys, 2017
10	20	Diagnostic criteria for vascular cognitive disorders: a VASCOG statement	Sachdev et al., 2014

Clustering analysis of references using CiteSpace yielded 71 clusters, with the top fifteen clusters depicted in Figure 9A. The modularity Q is 0.8421, and the weighted mean silhouette is 0.9383, indicating that the clusters are reasonable and convincing. Clusters #0 (diffusion-weighted imaging), #5 (magnetic resonance imaging), #9 (CT scan), and #13 (SPECT) are important neuroimaging methods concerning VCI. Besides, biomarkers, cerebral amyloid angiopathy, and cerebral blood flow are also considered research hotspots. References with a strong citation burst indicate significant attention and recognition from researchers within a specific timeframe. Gorelick PB’s study obtained the second-greatest citation burst strength (Figure 9B) and ranks third in co-citation counts. This article systematically synthesized the existing evidence, identified the current knowledge gap, and offered recommendations according to standard AHA criteria. It comprehensively covers definitions, diagnostic criteria, neuropathological and neuroimaging factors, cardiovascular risk factors, as well as treatment and prevention strategies related to VCI (Gorelick et al., 2011).

## 4 Discussion

### 4.1 Summary of basic information

With the continuous advancement in neuroimaging technologies, there is a growing number of studies exploring VCI neuroimaging. Quantitative analysis of papers included from 2000 to 2023 revealed an overall upward trend with some fluctuations in annual publications concerning VCI neuroimaging. This trend manifests an increasing scholarly focus on utilizing neuroimaging modalities to study VCI. Several stable groups of core authors have formed through the analysis of authors. Scheltens, Philip led the largest red cluster, while Na, Duk, L. and Kim, Sung Tae headed the blue and green clusters, respectively. Notably, the USA and China stand out as the most prolific contributors to publications concerning VCI neuroimaging. The Netherlands cooperated closely with other countries in different clusters, such as the USA, Germany, England, and Singapore. Nevertheless, there remains a need to bolster international collaboration for broader academic exchanges. Sungkyunkwan University was the most frequently published institution on the topic of VCI neuroimaging. *Stroke* and *Neurology* occupy central positions in the visualization map of journals and co-cited journals, and they are also ranked among the top three in publication counts and citations. This emphasizes their substantial role in advancing VCI neuroimaging research. Among the top ten journals, four of them are classified into the Q1 partition, and *the Journal of Neurology, Neurosurgery and Psychiatry* has the highest IF of 11.1. Moreover, eight of the top ten co-cited journals belong to the Q1 quartile, among which Lancet *Neurology* has the highest IF of 48. These journals may also provide options for researchers submitting manuscripts on VCI neuroimaging.

### 4.2 Research hotspots

Through the clustering analysis and citation burst of keywords and references, we can capture the research hotspots within the field, which will be discussed in the following aspects.

#### 4.2.1 Exploration of neuroimaging in cerebral small vessel disease

According to Figure 8, we can notice that small vessel disease has the largest citation burst strength, extending until 2023, and Figure 6B indicates that the keyword “small vessel disease” appears relatively later. Therefore, it is considered both the research hotspot and frontier. Cerebral small vessel disease (CSVD), a major cause of VCI, affects smaller cerebral vessels like arterioles, venules, and capillaries (Frantellizzi et al., 2020; Gao et al., 2022). The primary causes of CSVD encompass arteriolosclerosis, which is a result of age, hypertension, and other vascular risk factors, and cerebral amyloid angiopathy, which is caused by the accumulation of β-amyloid in the blood vessels. (Markus and de Leeuw, 2023)

Neuroimaging plays a crucial role in diagnosing and studying CSVD. Structural features on MRI encompass white matter hyperintensities (WMHs), cerebral microbleeds, lacunar infarcts, enlarged perivascular spaces, cortical superficial siderosis, and cerebral atrophy (Wardlaw et al., 2013a; Markus and de Leeuw, 2023). The MRI revealed a reduction in cerebral blood flow among patients with WMHs. (Appelman et al., 2008). And WMHs are often considered a sensitive marker for ischemic brain injury in elderly individuals. They appear as patchy areas with hyperintensity on T2-weighted and FLAIR sequences and as hypointense regions on T1-weighted imaging (Low et al., 2019). WMHs are correlated with decreased information processing speed and executive function. Additionally, it was reported that the baseline severity of WMHs is an independent predictor of VCI among patients with CSVD (Pavlovic et al., 2014). A meta-analysis also indicated that WMHs are associated with the increased occurrence of ischemic stroke, intracerebral hemorrhage, dementia, and mortality (Debette et al., 2019). Cerebral microbleeds (CMBs) present as focal deposits of hemosiderin caused by blood extravasation from damaged arterial walls. On T2-weighted imaging and SWI, they appear as small circular, or oval lesions with homogeneous signal intensity (Fazekas et al., 1999; Low et al., 2019). Wang et al. (2015) demonstrated that CMBs in stroke or transient ischemic attack patients are independently associated with cognitive impairment, particularly in attention. And this association seems to be led by strictly deep or lobar CMBs (Wang et al., 2015). Lacunar infarcts refer to circular or oval-shaped, subcortical, liquid-filled cavities with a diameter approximately between 3 mm and 15 mm. Patients with lacunar infarcts, especially multiple ones, are thought to have an increased risk of developing cognitive impairment and dementia. On FLAIR imaging, lacunes of presumed vascular origin usually display central hypointensity surrounded by hyperintense rims. These rims can also surround perivascular spaces when they pass through the WMH regions (Wardlaw et al., 2013b). Perivascular spaces are physiological spaces around small blood vessels when they pass through the brain parenchyma from the subarachnoid space (Markus and de Leeuw, 2023). As the main conduit for expelling interstitial fluid from brain tissues, perivascular spaces promote the exchange between cerebral fluid and interstitial fluid, clear the intracerebral waste, and maintain brain homeostasis (Yu et al., 2022). A prospective study involving 2612 participants revealed that enlarged perivascular spaces are associated with a significant decrease in information processing speed and increase the risk of VaD by more than four times (Ding et al., 2017). Cortical superficial siderosis represents the deposition of blood breakdown products occurring in the subarachnoid space or surface of the cerebral cortex, which is a key imaging feature of cerebral amyloid angiopathy. It’s prevalent among individuals with cognitive impairment, often linked to lower cognitive scores, WMHs, and microbleeds (Charidimou et al., 2015). Mok et al., 2011 indicated a direct correlation between cognitive deficits in white matter lesions and atrophy in the frontal lobe and global cortical gray matter.

Consequently, employing early neuroimaging examinations helps to detect and monitor disease, as well as offer guidance for treatment interventions, thereby preventing further damage to brain tissue and the progression of cognitive dysfunction.

#### 4.2.2 The diagnosis of VCI

The clinical diagnosis of VCI heavily relies on neuropsychological evaluations for cognitive disorders and neuroimaging findings that present evidence of clinical stroke or cerebral vascular injury (Gorelick et al., 2011). The cognitive decline associated with cerebrovascular diseases typically progresses slowly, affecting processing speed, complex attention, and executive function over time (Zanon Zotin et al., 2021). In addition, cognitive evaluations typically comprise five aspects: executive function, attention, memory, language, and visuospatial function. Neuroimaging provides important information on the neuroanatomical basis of VCI, playing a significant role in diagnosing and predicting VCI (Heiss et al., 2016). MRI is the gold-standard imaging method for diagnosing VCI, while CT is also an appropriate approach to diagnose the probable mild VCI or major VCI (VaD) in cases where only CT imaging is available. According to a guideline from the Vascular Impairment of Cognition Classification Consensus Study (VICCCS) in 2018, neuroimaging assessments primarily recommended estimating cerebral atrophy (atrophy and ventricular size, as well as medial temporal lobe atrophy), detecting WMHs using the age-related white matter changes scale, and assessing the number, size, and location of infarction and hemorrhage. Moreover, quantitative measurements of brain or WMHs volume can supplement these evaluations but are not considered primary for clinical diagnosis (Skrobot et al., 2018; Iadecola et al., 2019).

#### 4.2.3 The application of neuroimaging technologies in VCI

Neuroimaging is an important component of the assessment for patients with cognitive decline for the first time. Evidence for vascular pathology in the brain often relies on findings from CT or structural MRI, with the latter having higher sensitivity. In clinical practice, most acute stroke patients apply CT to exclude cerebral hemorrhage or other lesions, such as brain tumors. CT imaging also shows old stroke lesions and early signs of ischemia. Moreover, it has consistent superiority with MRI in identifying large infarcts and global atrophy, contributing to predicting subsequent cognitive dysfunction (Heiss et al., 2016; van der Flier et al., 2018). MRI is an ideal imaging method for assessing both brain structure and function as well as identifying cognitive deficits, providing a large amount of reliable information. T1-weighted, T2-weighted, and FLAIR sequences are utilized to detect brain atrophy, small and lacunar infarcts, as well as WMHs, respectively. As displayed in Figure 9A, #0 diffusion-weighted imaging (DWI) was the largest cluster, demonstrating that researchers tend to study VCI by applying DWI. DWI imaging provides image contrast based on the difference in diffusion amplitude of water molecules in the brain, which exhibits high sensitivity in the early detection of ischemic injury (Huisman, 2010). Furthermore, it differentiates acute from chronic ischemic injuries and identifies newly developed silent infarcts (Choi et al., 2000).

PET visualizes metabolism and protein deposition based on the application of radioactive tracers. Neuron activity correlates with glucose metabolism, and cerebral hypometabolism detected by ^18^F-FDG-PET serves as a marker of neurodegeneration (Chételat et al., 2020). Furthermore, measuring regional cerebral glucose metabolism distinguishes different types of cognitive impairment with low metabolic patterns. In VCI, hypometabolism predominantly manifests in subcortical regions and the primary sensorimotor cortex, with a comparatively lesser impact on association areas in contrast to AD (Heiss et al., 2016). Memory function in subcortical stroke patients with cognitive dysfunction demonstrates an association with prefrontal lobe metabolism. In AD patients, memory ability correlates with left hippocampus and temporal lobe metabolism (Reed et al., 2000). Amyloid plaques are typical histopathological manifestations in AD, and their increased deposition can be assessed using various PET-tracers, such as 11C-Pittsburgh compound B (Heiss and Zimmermann-Meinzingen, 2012). The continuous exploration of PET-tracers is beneficial for deepening the understanding of pathophysiology, facilitating early diagnosis, and subsequently providing insights for treatment and management. SPECT imaging is primarily applied to quantify cerebral perfusion. According to the European Federation of Neurological Societies (EFNS) guideline, SPECT can serve as a supplementary tool in differentiating various types of dementia in cases of uncertain diagnoses (Hort et al., 2010). In conclusion, the field of neuroimaging is undergoing rapid evolution, and future technological advancements may provide stronger support for enhancing the diagnostic accuracy of VCI and offer inspiration for exploring treatment targets.

### 4.3 Research frontiers

As shown in Figure 8, keywords including small vessel disease, connectivity, biomarkers, and lacunar stroke have acquired the recent citation burst, indicating possible future research directions related to VCI neuroimaging. Additionally, cerebral small vessel disease has already been discussed in Part 4.2.1.

#### 4.3.1 Functional and structural connectivity

The human brain is a complex, interconnected network where nodes are linked to facilitate efficient information processing and integration (de Reus et al., 2014). The effects of vascular lesions on cognitive function are mediated through alterations in functional and structural connectivity. Functional MRI and electrophysiological techniques can measure functional connectivity, while structural connectivity is evaluated through diffusion imaging (Dichgans and Leys, 2017). Li et al., 2021 indicated that mild cognitive impairment was related to disrupted functional brain networks, and the functional connectivity strength in the middle temporal gyrus was obviously decreased. Moreover, disrupted functional connectivity between the frontal lobe and middle temporal gyrus was detected in the vascular mild cognitive impairment patients, which may be associated with executive dysfunction. The structural connectivity of the brain network facilitates predicting the occurrence of dementia and differentiating the diagnosis of dementia. White matter network disruption plays an important role in the progression to dementia for elderly patients with SVD, and structural network efficiency may serve as an early predictor of dementia (Tuladhar et al., 2016). Feng et al., 2021 observed decreased structural connections in the frontal-prefrontal, frontal-subcortical, and prefrontal-subcortical regions among patients with SIVD. As for AD patients, the structural connectivity in the frontal and prefrontal regions increased, while it decreased in the temporal and occipital regions.

#### 4.3.2 Neuroimaging biomarkers

With the advances in neuroimaging technologies, neuroimaging biomarkers are playing a significant role in the process of achieving precision medicine. Structural MRI analysis revealed that volume and symmetry differences in the hippocampus, amygdala, nucleus accumbens, and globus pallidus may help to distinguish VaD from AD. Additionally, this combination of different structural MRI biomarkers provides more information than a single biomarker and facilitates better differential diagnosis (Zheng et al., 2019). Brain network features show great advantages in explaining cognitive impairment. In patients with subcortical ischemic vascular disease, cognitive dysfunction appears closely related to the microstructural changes of multiple white matter fibers connecting diverse cortical and subcortical regions. A study indicated that damage to white matter integrity occurs in the preclinical stage of VCI, and diffusion tensor imaging could distinguish various stages of VCI arising from CSVD (Du et al., 2021). Detection of brain networks using resting-state functional MRI revealed disrupted global network topology with obviously increased path length and modularity in patients with subcortical vascular mild cognitive impairment. Moreover, enhanced intermodule connectivity in the inferior and superior parietal gyrus correlates with poor cognitive function (Yi et al., 2015). Furthermore, PET provides quantitative measurements of brain function. When employing FDG-PET to assess regional cerebral glucose metabolism, hypometabolic patterns in the thalamus, brainstem, and cerebellum were observed in VaD patients. This differs from the typical metabolic pattern seen in AD patients, manifesting as hypometabolism in the posterior cingulate and temporal-parietal cortex (Nestor et al., 2018). In conclusion, it requires more neuroimaging biomarkers with specificity and sensitivity to promote early identification and intervention of VCI in clinical practice.

#### 4.3.3 Lacunar stroke

Lacunar stroke refers to small subcortical infarcts resulting from ischemia in the deep perforating arterial region of the brain. It has been regarded as a strong predictor of post-stroke dementia in a study covering a period of 24 years (Béjot et al., 2011). Lacunar stroke mainly encompasses two terms: recent small subcortical infarcts (RSSI) and lacunes of presumed vascular origin. MRI stands as a conventional imaging technique for identifying lacunar stroke. DWI is considered sensitive and specific in detecting acute lacunar stroke, while T2-weighted and FLAIR sequences were superior to CT in identifying chronic lacunes. Besides, 7T ultra-high-field MRI provides a promising method to determine the characteristics of the perforating arterial wall in the pathogenesis of lacunar stroke, which is expected to improve the ability of detection and diagnosis for lacunar stroke (Jiang et al., 2021).

### 4.4 Study limitations

This bibliometric analysis still has certain limitations. Firstly, our literature retrieval was limited to the Core Collection of the Web of Science database because it provides complete information beneficial for the data analysis of CiteSpace and VOSviewer. However, this approach might have excluded articles available in other databases. Secondly, we only included literature published in English, potentially leading to selective bias in article screening. Finally, no distinction was made regarding the quality of the included literature when performing this bibliometric analysis, and the lower-quality articles could impact the quality of this study.

## 5 Conclusion

To the best of our knowledge, this is the first comprehensive and systematic bibliometric analysis of VCI neuroimaging, spanning from 2000 to 2023. After the comprehensive retrieval of relevant literature, we conducted a visualized analysis to explore the current research status, research hotspots, and possible future research trends in VCI neuroimaging. The author, country, institution, and journal that published the most articles were Na, Duk L., the USA, Sungkyunkwan University, and the *Journal of the Neurological Sciences*, respectively. Notably, dementia, cognitive impairment, magnetic resonance imaging, small vessel disease, and stroke are the most frequently appearing keywords. Through the analysis of keywords and references, the research hotspots encompass the relationship between the neuroimaging of CSVD and VCI, the diagnosis of VCI, and neuroimaging approaches for VCI. In addition, future research directions involve investigating CSVD, functional and structural connectivity, neuroimaging biomarkers, and lacunar stroke.

## Data availability statement

The original contributions presented in this study are included in the article/Supplementary material, further inquiries can be directed to the corresponding authors.

## Author contributions

FX: Conceptualization, Data curation, Formal analysis, Writing – original draft. ZD: Formal analysis, Software, Writing – original draft, Data curation. WZ: Methodology, Supervision, Writing – review and editing. YY: Data curation, Formal analysis, Software, Writing – review and editing. FD: Data curation, Formal analysis, Writing – review and editing. PH: Methodology, Supervision, Writing – review and editing. HC: Conceptualization, Funding acquisition, Supervision, Writing – review and editing.
