# Supplementary material for: Exploring research hotspots and emerging trends in neuroimaging of vascular cognitive impairment: a bibliometric and visualized analysis
Source: Front Aging Neurosci. 2024 Jul 8;16:1408336. doi: 10.3389/fnagi.2024.1408336 (PMC11260638; doi:10.3389/fnagi.2024.1408336)
Supplement: Supplementary file 1 [file Table_1.DOCX]

**Supplementary Table 1** The search strategy of VCI neuroimaging.

| Number | Search terms |
| --- | --- |
| #1 | TS=(“Neuroimaging” OR “Structural magnetic resonance imaging” OR “sMRI” OR “Functional magnetic resonance imaging” OR “fMRI” OR “Magnetic resonance imaging” OR “MRI” OR “Diffusion weighted imaging” OR “DWI” OR “Diffusion tensor imaging” OR “DTI” OR “Magnetic resonance spectroscopy” OR “MRS” OR “Magnetic resonance* ” OR “Positron emission tomography” OR “PET” OR “Computed tomography” OR “CT” OR “Single photon emission computed tomography” OR “SPECT” OR “Functional near-infrared spectroscopy” OR “fNIRS” OR “Electroencephalogra* ” OR “EEG” OR “Magnetoencephalogra* ” OR “MEG” ) |
| #2 | TS=(“Vascular cognitive impairment” OR “Vascular mild cognitive impairment” OR “Vascular cognitive* ” OR “Vascular dementia” OR “Subcortical vascular dementia” OR “Subcortical ischemic vascular dementia” OR “Arteriosclerotic dementia” OR “Chronic progressive subcortical encephalopathy” OR “Binswanger encephalopathy” OR “Subcortical leukoencephalopathy” OR “Binswanger disease” OR “Binswanger's disease” OR “Binswangers disease” OR “Subcortical leukoencephalopathies” OR “Subcortical arteriosclerotic encephalopathy” OR “Subcortical arteriosclerotic encephalopathies” OR “Binswanger's encephalopathy”) |
| #3 | #1 AND #2 |
